# Study on Wear Characteristics of Revolute Clearance Joints in Mechanical Systems

**DOI:** 10.3390/mi13071018

**Published:** 2022-06-27

**Authors:** Zhengfeng Bai, Zhiyuan Ning, Junsheng Zhou

**Affiliations:** 1 Department of Mechanical Engineering, Harbin Institute of Technology, Weihai 264209, China; zhoujunsheng1@hotwater.com.cn; 2 Department of Astronautics Engineering, Harbin Institute of Technology, Harbin 150001, China; 20b918089@stu.hit.edu.cn

**Keywords:** clearance joint, contact and friction, wear, planar mechanism, dynamics characteristics

## Abstract

The existence of clearance causes contact-impact forces in joints, which lead to surface wear and incessant material loss of the joint surface during the motion of mechanisms. In this work, the wear characteristics of dry revolute clearance joints in planar mechanisms are studied using a computational methodology. The normal contact force model and the tangential friction force model are established to describe the contact-impact in clearance joints. Then, the dynamic wear model based on the Archard’s wear model is established to predict the wear characteristics of clearance joints in mechanisms. The dynamic wear depths of clearance joints are obtained in two steps. The first step is the dynamics analysis of mechanisms to obtain the contact and sliding characteristics between the bearing and journal in the clearance joint. The second step is the dynamic wear depth analysis of clearance joints based on dynamic Archard’s wear model. Finally, a planar slider-crank mechanism with two revolute clearance joints between the connecting rod and its adjacent links is used as the implement example. Different case studies are performed to investigate the wear characteristics of clearance joints in mechanical systems.

## 1. Introduction

Clearance in joints of mechanisms is unavoidable due to assemblage, manufacturing errors and wear. Joint clearance reduces the motion accuracy, causes vibration, induces joint wear and affects the dynamic performances of mechanical systems. In the past decades, many works of studying the dynamic responses of mechanisms with clearances analytically and experimentally have been implemented [1,2,3,4,5,6,7,8,9,10].

Khemili and Romdhane [11] presented an investigation on dynamics modeling and analysis of a slider-crank mechanism considering a planar revolute clearance joint based on ADAMS software and experimental tests. Erkaya et al. [12] presented a study on decreasing deviations arising from a clearance joint in planar linkage mechanisms by a neural network-genetic algorithm procedure. Muvengei et al. [13] investigated dynamics and motion modes of a slider-crank mechanism with two planar revolute clearance joints. Bai and Sun [14] studied dynamic responses of a planar mechanism system including three planar revolute clearance joints. The effects of multi-clearance joints on dynamic characteristics are discussed. Wang et al. [15] presented a non-penetration approach of frictional contact analysis for modeling revolute clearance joints of planar rigid multibody mechanical systems. Tan et al. [16] investigated effects of friction on dynamic behavior of a planar slider-crankmechanism considering revolute joints with radial clearance using the LuGre friction model. Liu and Bai [17] investigated clearance effects on dynamic responses of a space robot manipulator with planar revolute clearance joints between manipulator arms. Salahshoor et al. [18] studied the effect of joint stiffness on vibration behaviors of a typical slider-crank mechanism with a flexible component and joint clearances. Zhan et al. [19] presented a unified motion reliability analysis method for general planar parallel manipulators of PPMs with interval clearance variables of revolute and prismatic joints. Two typical types of PPMs are presented to perform analysis. Also, recently, researchers have been focused on the effects of 3D revolute clearance joints [20,21,22,23]. All of the research indicated that clearance leads to contact and impact in joints, which will lead to significant effects on dynamic responses of mechanical systems. 

Consequently, the contact and impact forces in clearance joints will lead to surface wear and incessant material loss of joint elements during the motion of mechanisms. Mukras [24,25,26] investigated the wear characteristics of clearance joints theoretically and experimentally. The wear analysis was based on the Archard wear model using a nonlinear finite element analysis method. The dynamic wear phenomenon of clearance joint in slider-crank mechanisms was investigated for different clearance shapes and sizes. Zhao et al. [27] studied the wear characteristics of revolute clearance joints in flexible multibody systems. The contact force in the clearance joint was applied using the continuous contact force model proposed by Lankanrani and Nikravesh, and the friction effect is considered using the LuGre friction model. A flexible planar slider-crank mechanism demonstrates the investigation. Lai et al. [28] studied the revolute joint clearance wear in the low-velocity planar mechanism using computational and experimental methods. Flores [29] studied the wear phenomena of revolute clearance joints using a computational method. The contact force model in the clearance joint was established using the contact force model proposed by Lankanrani and Nikravesh. The results indicated that the wear in clearance joints is non-regular. The wear in joints enlarges the clearance size. Ordiz [30] proposed a method to study the effect of joint clearances on the fatigue life of machines, in which the increase in dynamic loads due to wear in clearances was considered. Results showed that clearances may have a great impact in the service life of machines. However, most of the previous investigations focused on clearance effects on the dynamic performance of mechanisms. Limited works have been focused on the dynamic wear characteristics of clearance joints in mechanisms. In fact, wear changes the clearance size of joints and affects dynamic performance of mechanisms. Wear causes the precision of mechanism to reduce, especially for high-accuracy and long-life mechanisms. Less research and models have been presented to show how wear in clearance joints of mechanical systems is important. 

In this work, the main objective is to study the wear phenomenon of dry revolute clearance joints in mechanisms using a computational methodology. Case studies for different effect factors are performed to investigate the dynamic wear characteristics of clearance joints. The normal contact force model and the tangential friction force model are established to describe the contact-impact forces in clearance joints. Dynamic Archard’s wear model is established to predict the dynamic wear depth of clearance joints in mechanisms. First, dynamics characteristics of mechanisms are analyzed. The contact and sliding characteristics between the journal and bearing in clearance joints are obtained. Then, the dynamic wear depths of clearance joints are analyzed based on the dynamic Archard’s wear model. Finally, a planar slider-crank mechanism with two revolute clearance joints is used as the implement example to perform the investigation. The dynamic wear characteristics of clearance joints in mechanical systems with multi-clearance joints are presented and discussed.

## 2. Contact Force Model in Revolute Clearance Joint

**Figure 1:**
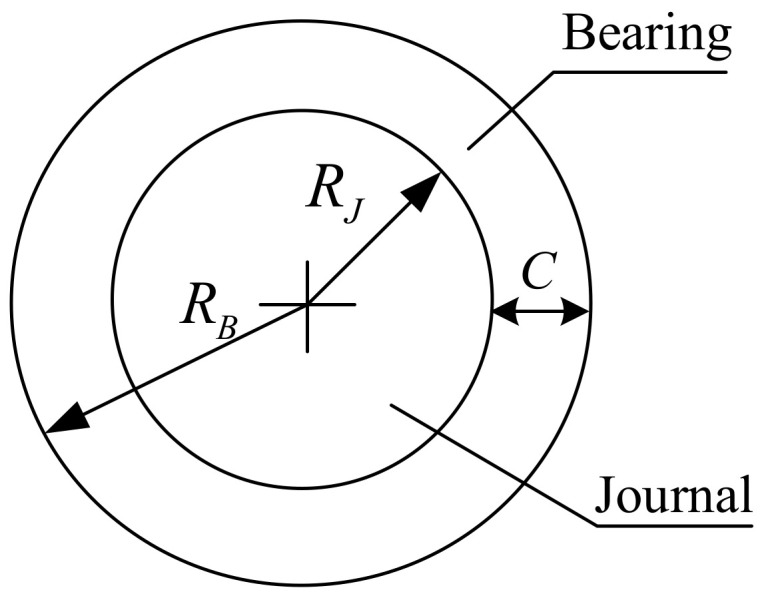
Schematic of revolute joint with clearance.

Figure 1 depicts a revolute joint with clearance. The radius of bearing and journal in the revolute clearance joint are $R_{B}$ and $R_{J}$, respectively. The difference between their radii is defined as the radial clearance. Thus, the radial clearance is expressed as Equation (1):

(1) $$c=R_{B}-R_{J}$$

**Figure 2:**
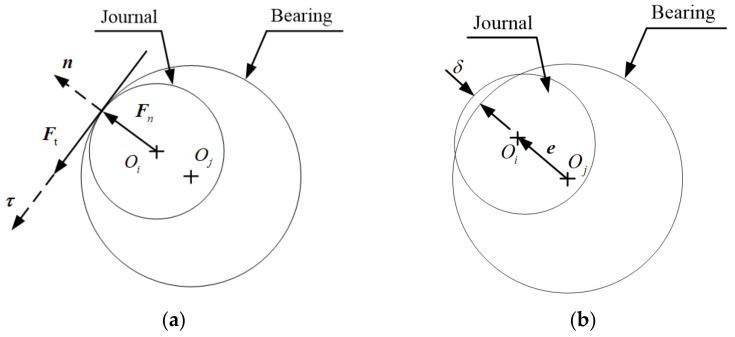
Contact in revolute clearance joint. (**a**) contact forces; (**b**) contact deformation.

Usually, there are contact-impact characteristics in a real joint with clearance in mechanisms, which includes normal contact force and tangential friction force, shown in Figure 2. When the journal and bearing are in contact, a contact force is applied perpendicular to the plane of contact. The contact forces between the two elements of clearance joints can be described as normal contact force, $F_{n}$, and tangential contact force, $F_{t}$. Therefore, the constraints of clearance joint are modeled as contact force constraints, which is in line with the real joint. The contact deformation caused by contact and impact between bearing and journal can be represented as Equation (2):

(2) δ=e−c

where *c* is the clearance size and *e* is the eccentricity of the journal center relative to the bearing center, as shown in Figure 2b.

Consequently, to evaluate contact forces efficiently between the bearing and the journal for revolute joints with clearance, special attention must be paid to the numerical description of the contact force model [31,32,33,34,35,36]. In this work, the normal contact between the journal and bearing in a revolute clearance joint is established using the Lankarani and Nikravesh contact force model, which is based on the Hertz model with a damping term and it is expressed as Equation (3) [36]:

(3) $$F_{n}=K_{n}\delta^{n}+D\dot{\delta }$$

where δ is the deformation, $\dot{\delta }$ is the relative deformation velocity. D is the nonlinear damping coefficient. Nonlinear damping coefficient D can be expressed as Equation (4):

(4) $$D=\eta \delta^{n}$$

where η is the viscous damping factor. It can be expressed as Equation (5):

(5) $$\eta =\frac{3K_{n}\left(1-c_{e}^{2}\right)}{4{\dot{\delta }}^{\left(-\right)}}$$

where ${\dot{\delta }}^{\left(-\right)}$ is the initial relative velocity of the impact point., $c_{e}$ is the material recovery coefficient.

Further, D can be expressed as Equation (6):

(6) $$D=\frac{3K_{n}\left(1-c_{e}^{2}\right)\delta^{n}}{4{\dot{\delta }}^{\left(-\right)}}$$

Consequently, the normal contact force $F_{n}$ can be expressed as Equation (7):

(7) $$F_{n}=K_{n}\delta^{n}\left[1+\frac{3(1-c_{e}^{2})\dot{\delta }}{4{\dot{\delta }}^{\left(-\right)}}\right]$$

The tangential contact of clearance joints is represented using the friction force model. The best-known friction model is Coulomb friction model. However, there will be errors in numerical calculation when relative tangential velocity is close to zero. Here, a modified Coulomb friction model with a dynamic friction coefficient is used for tangential contact of clearance joints, which can avoid numerical difficulties. The expression of the modified Coulomb friction model is shown as Equation (8) [8,33]:

(8) $$F_{t}=-\mu (v_{t})F_{n}\frac{v_{t}}{\left|v_{t}\right|}$$

where $v_{t}$ is the relative sliding velocity in the contact point. The dynamic friction coefficient $\mu (v_{t})$ is a function of tangential sliding velocity and expressed as Equation (9):

(9) $$\mu (v_{t})=\{\begin{array}{cc} \;-\mu_{d}sign(v_{t}) & for\;|v_{t}|>v_{d}\\ -\{\mu_{d}+(\mu_{s}-\mu_{d}){\left(\frac{|v_{t}|-v_{d}}{v_{s}-v_{d}}\right)}^{2}[3-2(\frac{|v_{t}|-v_{d}}{v_{s}-v_{d}})]\}sign(v_{t}) & \;\;for\;v_{s}\leq |v_{t}|\leq v_{d}\\ \;\mu_{s}-2\mu_{s}{\left(\frac{v_{t}+v_{s}}{2v_{s}}\right)}^{2}(3-\frac{v_{t}+v_{s}}{v_{s}}) & for\;|v_{t}|<v_{s} \end{array}$$

where $\mu_{s}$ is the static friction coefficient. $\mu_{d}$ is the critical velocity of the maximum dynamic friction. $v_{s}$ is the critical velocity of static friction. $v_{d}$ is the critical velocity of the maximum dynamic friction.

## 3. Dynamics Equations of Mechanism Systems with Clearances

The dynamics equations of the mechanism system are established considering the clearance model. The clearance leads to two different motion phases of bodies connected with the clearance joint: one is where the bodies move freely in the clearance and the other is where the bodies contact and interact. Therefore, the mechanism system with clearance between bodies is a variable topology system, which is solved using a dynamic segmentation modeling method. Further, the dynamics equation is obtained using the Lagrange multiplier method and the dynamic equations are formulated as Equations (10) and (11) [8].

In the free-motion phase, the dynamics equation is presented as:

(10) $$\begin{array}{l} M\ddot{q}+C\dot{q}+Kq+\Phi_{q}^{T}\lambda =F\\ \Phi (q,t)=0 \end{array}$$

where q is the generalized coordinate column matrix, M, C and K are the generalized mass matrix, generalized damp matrix and generalized stiffness matrix, respectively. $\Phi_{q}$ is the Jacobin matrix of the constraint equation. F is the generalized force matrix. λ is the Lagrange multiplier column matrix.

In the contact phase, the bodies interact and the contact forces exist in the clearance between the bearing and journal. The dynamics equation is presented as:

(11) $$\begin{array}{l} M\ddot{q}+C\dot{q}+Kq+\Phi_{q}^{T}\lambda =F+F_{c}\\ \Phi (q,t)=0 \end{array}$$

where $F_{c}$ is the contact force relative to the q, which contains both normal contact force, $F_{n}$, expressed as Equation (7), and tangential friction force, $F_{t}$, expressed as Equation (8).

## 4. Dynamic Wear Model of Revolute Clearance Joint

In a case of a revolute clearance joint in a mechanism, wear would occur when the journal and bearing of the clearance joint contact with relative motion. In the numerical modeling and analysis of clearance joints in a mechanism, Archard’s wear model is usually used, which correlates the wear volume with some physical and geometrical properties of sliding bodies, such as applied load, sliding distance and hardness. In this work, Archard’s wear model is used to calculate the wear amount of revolute clearance joints in mechanical systems. This model was developed and based on experimental tests and can be expressed by [37],

(12) $$\frac{V}{s}=K\frac{F_{n}}{H}$$

where V is the wear volume, s represents the sliding distance, K is the dimensionless wear coefficient, $F_{n}$ represents the normal contact force and H is the hardness of the softer material.

This formula shows the relationship between the wear volume and the relative sliding distance, normal contact force as well the hardness of softer materials. In real engineering, the wear depth of the joint is widely used. In order to obtain the wear depth during the wear process, assume that the actual contact area is A, Archard’s wear model of Equation (12) can be written as Equation (13):

(13) $$\frac{V}{As}=\frac{h}{s}=kp$$

where h is the wear depth; p is the contact pressure, which is expressed as $p=\frac{F_{n}}{A}$. k is the linear wear coefficient and is expressed as $k=\frac{K}{H}$.

In the motion of mechanisms with clearance joints, the contact point of joints keeps changing, and the contact force and sliding distance are different in different moments. It means the sliding distance and contact stress between the journal and bearing changes with time during the motion of mechanism. The wear process is considered to be a dynamic process. Further, Archard’s wear model of Equation (13) is expressed as the following differential form in Equation (14):

(14) $$\frac{dh}{ds}=kp$$

Because the sliding distance between journal and bearing is always changing with the motion of mechanical system, the sliding speed of the contact point which is easier to measure is used to calculate the sliding distance. Consequently, the dynamic wear depth in Equation (14) is expressed as Equation (15):

(15) dh=kpvdt

where v is the sliding speed.

The sliding velocity *v* can be obtained by the rotational speed between journal and bearing in the clearance joints. the rotational speed of the journal relative to the bearing is expressed as Equation (16):

(16) $$\omega_{12}=\omega_{1}\mp \omega_{2}$$

where $\omega_{1}$ and $\omega_{2}$ are the rotational speeds of bearing and journal, respectively. The sign is positive when $\omega_{1}$ and $\omega_{2}$ are rotating in different directions and the sign is negative when $\omega_{1}$ and $\omega_{2}$ are rotating in the same direction.

Further, the sliding speed *v* at contact point between journal and bearing is expressed as Equation (17):

(17) $$v=(R_{J}+\delta )\omega_{12}=(R_{J}+\delta )(\omega_{1}\mp \omega_{2})$$

where $R_{J}$ is the diameter of the journal.

Further, for any given time *t*, the wear depth at contact points of revolute clearance joints is calculated as Equation (18):

(18) $$h=\int_{0}^{t}kpvdt$$

## 5. Computational Strategy of Wear Analysis for Clearance Joints

In order to analyze the wear characteristics in clearance joints of mechanisms, two main steps are presented, which are: dynamics analysis of mechanisms with clearances, and wear analysis of clearance joints. Firstly, the dynamics characteristics of mechanisms with clearances are analyzed. The contact forces of clearance joints and the sliding velocity between the journal and bearing are obtained. Secondly, the wear depth of clearance joints is calculated based on the dynamic wear model of Equation (18).

**Figure 3:**
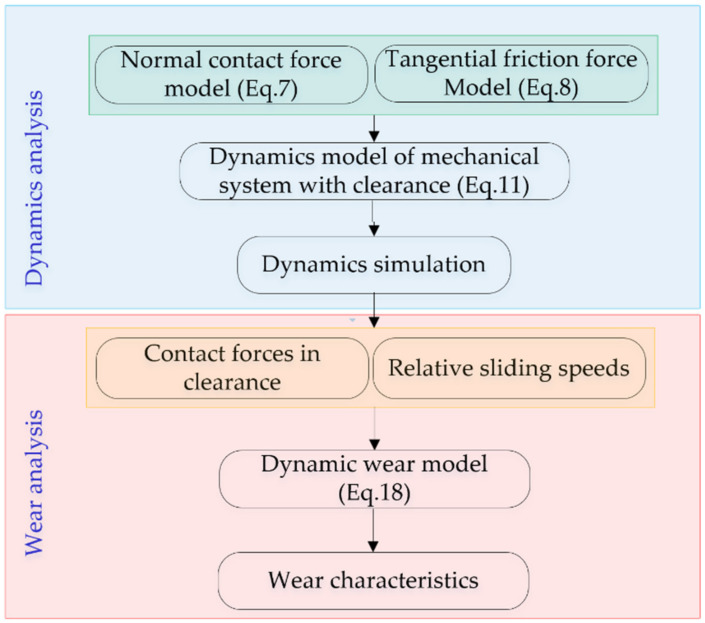
Computational process of clearance joint wear in mechanical systems.

The detailed wear analysis process is shown as Figure 3 and performed as following:

(1) Establish the contact force models of the clearance joint, including the normal contact force model and the tangential friction force model;
(2) Establish the dynamics model and perform the dynamic simulation of mechanical system with joint clearances;
(3) Dynamics analysis and draw the contact force in the clearance joint as well as the relative sliding speeds between journal and bearing of the clearance joint;
(4) Calculate the wear depth of the clearance joint based on the dynamic Archard’s wear model.
(5) Analyze and discuss the wear characteristics of the clearance joint for mechanisms in different case studies.

## 6. Numerical Example and Results

### 6.1. Numerical Example: Planar Slider-Crank Mechanism with Multi-Clearance Joints

**Figure 4:**
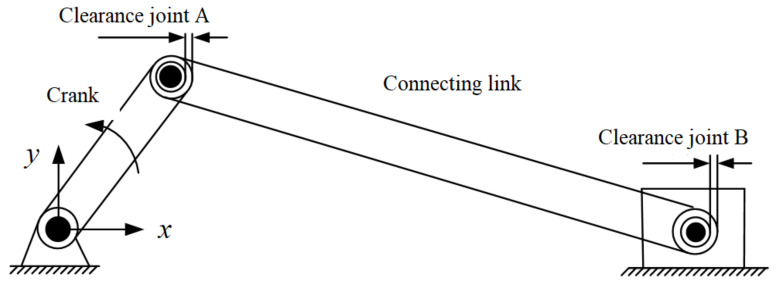
Slider–crank mechanism with multi-clearances.

In this section, a planar slider-crank mechanism with two revolute clearance joints is used to represent the investigation. Figure 4 depicts the configuration of the planar slider-crank mechanism, in which two revolute clearance joints exist between the connecting rod and the slider as well as the crank. They can also be called revolute joint A and revolute joint B. The connecting rod is considered as a flexible link, and the crank and slider are considered as rigid bodies.

The crank is the driving link. The length and inertia properties of the slider-crank mechanism components are listed in Table 1 and the parameters used in the dynamic simulations are presented in Table 2.

### 6.2. Results of Wear Characteristics

In the dynamic simulation, the crank is the driving link and the speed of crank is 200 rpm. The initial clearance size is 0.5 mm for each clearance joint, which are revolute joint A and revolute joint B. Here, two case studies for different numbers of revolute clearance joints are considered in the simulation. In the first case study, there is only one clearance joint. Revolute joint A is considered as an imperfect joint with clearance, and revolute joint B is considered as an ideal joint without clearance. In the second case study, there are two clearance joints. Both revolute joint A and revolute joint B are considered clearance joints. Long-time simulations are performed and the results presented below are plotted against two full crank rotations after a steady state has been reached.

**Figure 5:**
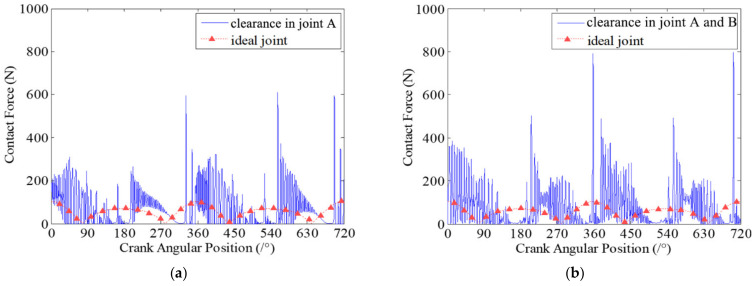
Contact forces in revolute clearance joint A. (**a**) case 1: there is one clearance joint in the mechanism; (**b**) Case 2: there are two clearance joints in the mechanism.

**Figure 6:**
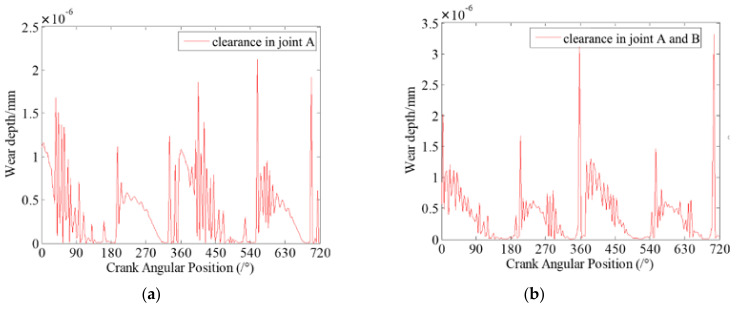
Dynamic wear depth in revolute clearance joint A. (**a**) case 1: there is one clearance joint in the mechanism; (**b**) Case 2: there are two clearance joints in the mechanism.

Figure 5 presents the contact forces in revolute clearance joint A for the two case studies. The contact forces in clearance joints are compared with the reaction forces in ideal joints. It clearly shows that the contact forces in clearance joints are much higher than that in ideal joints. During the motion of mechanism, the contact and impact between journal and bearing in clearance joints are oscillating obviously, with high frequency and peaks. Besides, the amplitudes of contact and impact forces are very large. When two clearance joints are considered in the mechanism, the vibration peaks of the contact forces increase compared with contact forces from the case where one clearance joint is considered. It is due to the two clearance joints interacting with each other. Further, the increased contact forces in clearance joints cause more severe wear. Figure 6 shows the dynamic wear depth of clearance joint A. It indicates that the wear depth of clearance joints is different in each crank motion position. The wear amount is dynamically changing during the motion of the mechanism. The wear of revolute clearance joints is not uniform, but more severe in some regions of crank position. Also, the wear depth in revolute clearance joints shows similar characteristics of contact forces. Additionally, it shows that the dynamic wear depth is larger when considering two clearances compared with the wear depth when only one clearance joint is considered in the mechanism.

## 7. Parametric Effects on Clearance Joint Wear

In this section, the effects of different factors on clearance joint wear are analyzed. Three factors of clearance size, contact stiffness and driving speed are presented. For each factor, four case studies are compared and discussed, which are listed in Table 3. 

### 7.1. Effect of Clearance Size on Wear Characteristics

**Figure 7:**
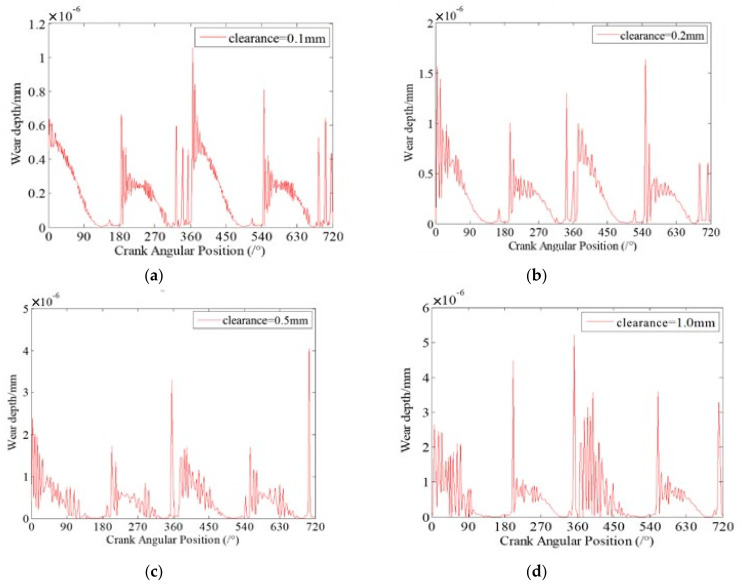
Dynamic wear depth in revolute clearance joint A for different clearance sizes. (**a**) 0.1 mm; (**b**) 0.2 mm; (**c**) 0.5 mm; (**d**) 1 mm.

Clearance size is one of the key factors that affect the dynamics characteristics and wear characteristics of clearance joints in mechanism. In this section, the effects of different clearance sizes on dynamic wear characteristics of clearance joints in the slider-crank mechanism are investigated. Four case studies with different clearance sizes are presented. The clearances exist in revolute joint A connecting the crank and connecting rod, and revolute joint B connecting the slider and the connecting rod. The sizes of each clearance are 0.1 mm, 0.2 mm, 0.5 mm and 1 mm. The dynamic wear depths at clearance joint A for different clearance sizes are presented in Figure 7.

**Figure 8:**
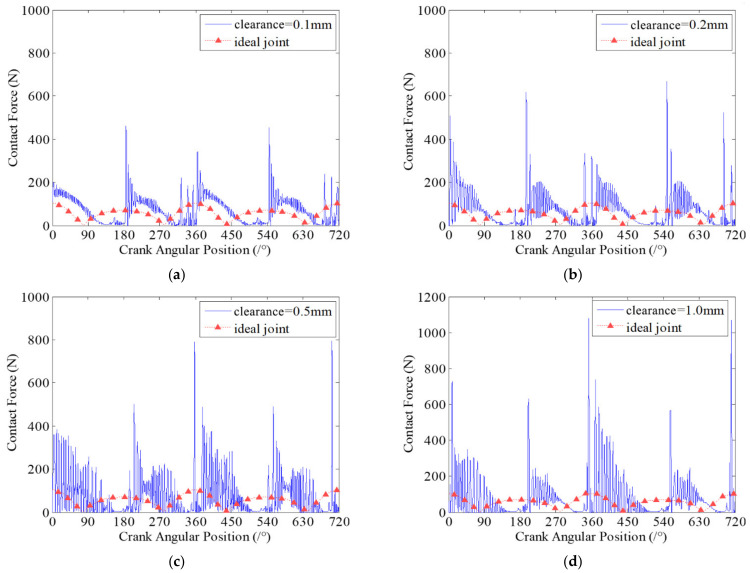
Contact forces in revolute clearance joint A for different clearance sizes. (**a**) 0.1 mm; (**b**) 0.2 mm; (**c**) 0.5 mm; (**d**) 1 mm.

It can be seen from Figure 7 that the dynamic wear depths of clearance joint A are changing with the motion of the mechanism, which indicates that the clearance joint exhibits non-uniform wear during one motion cycle of the mechanism. Comparing the four case studies with different clearance sizes, it clearly shows that different clearance sizes present similar dynamic wear phenomenon, but the wear depths of clearance joints are obviously different. When the clearance size is smaller, the wear level is weaker, and the maximum wear depth is smaller. As the clearance size increases, the wear level is more severe and the maximum wear depth increases. When clearance size is 1 mm, the maximum wear depth is about 3.2 times the wear depth when clearance size is 0.2 mm. The reason is that as the clearance size increases, the journal and bearing contact and impact severely, causing a larger contact force in the clearance joint, as shown in Figure 8. The contact forces in clearance joint are one of the most important factors causing wear. Therefore, the clearance size is one important factor affecting the wear characteristics of clearance joints in mechanisms, which cannot be ignored and must be strictly controlled in the design of mechanisms.

### 7.2. Effect of Contact Stiffness on Wear Characteristics

**Figure 9:**
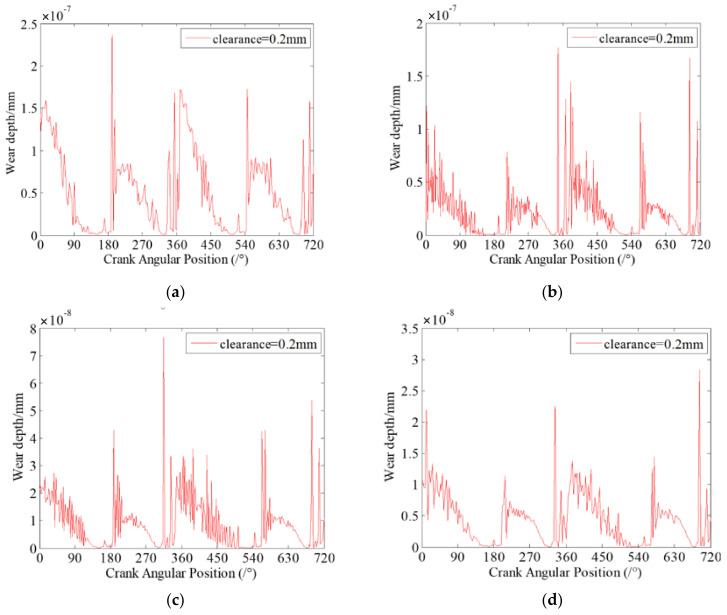
Dynamic wear depth in revolute clearance joint A for different cases. (**a**) 1 × 10^6^ N/mm; (**b**) 5 × 10^6^ N/mm; (**c**) 1 × 10^7^ N/mm; (**d**) 5 × 10^7^ N/mm.

**Figure 10:**
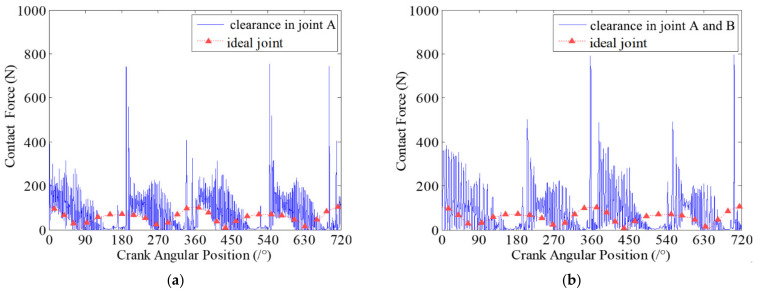

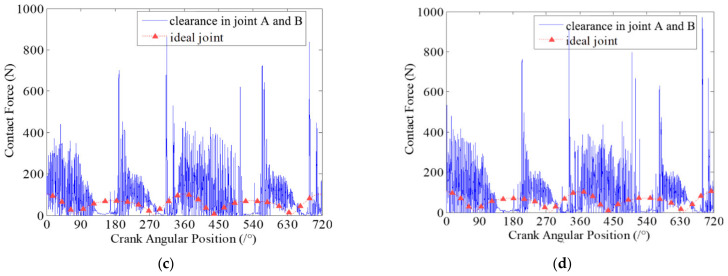
Contact forces in revolute clearance joint A for different cases. (**a**) 1 × 10^6^ N/mm; (**b**) 5 × 10^6^ N/mm; (**c**) 1 × 10^7^ N/mm; (**d**) 5 × 10^7^ N/mm.

When the journal and bearing in clearance joints contact and impact, the contact stiffness of the two contact elements will be an important factor on the contact and impact forces in clearance joints. Therefore, the contact stiffness coefficient has significant effects on the wear characteristics of clearance joints in mechanisms. In this section, four case studies with different contact stiffness coefficients are presented to investigate the effects of contact stiffness on the dynamic wear characteristics of clearance joints. Similarly, assuming there are two clearance joints, one of them is set in revolute joint A connecting the crank and connecting rod, and another is set in revolute joint B connecting the slider and connecting rod. The clearance size is 0.2 mm and the driving speed of crank is 200 rpm. Four case studies for different contact stiffness coefficients are investigated and the contact stiffness coefficients are considered as 1 × 10^6^ N/mm, 5 × 10^6^ N/mm, 1 × 10^7^ N/mm and 5 × 10^7^ N/mm, respectively. The dynamic wear depths and the contact forces at clearance joint A for different case studies are presented in Figure 9 and Figure 10, respectively.

Figure 9 shows that a larger contact stiffness coefficient can effectively reduce the wear level between contact surfaces of clearance joints. As the contact stiffness coefficient increases, the maximum wear depth of the clearance joint decreases, as shown in Figure 9, but the contact forces increase, as shown in Figure 10. It is due to the fact that as the contact stiffness coefficient increases, the ability of joint elements to resist deformation is stronger, and the contact deformation between journal and bearing decreases. Therefore, it can effectively reduce the wear level between contact surfaces of clearance joints. When the contact stiffness coefficient increases from $1\times 10^{6}$ N/mm to $5\times 10^{7}$ N/mm, the maximum wear depth is reduced approximately ten times. Although the contact forces between journal and bearing are increased as the contact stiffness coefficient increases, the maximum wear depth of clearance joint decreases.

### 7.3. Effect of Driving Speeds on Wear Characteristics

**Figure 11:**
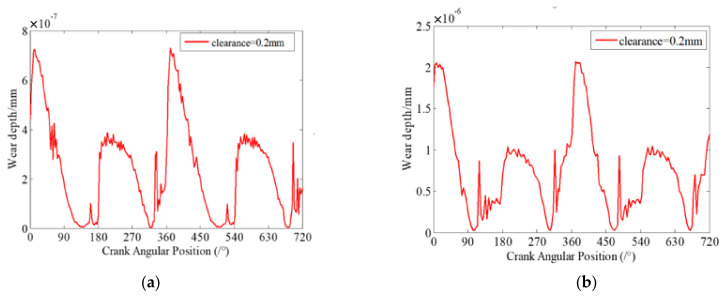

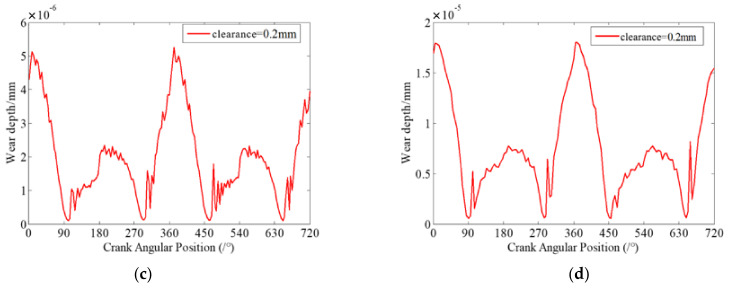
Dynamic wear depth in revolute clearance joint A for different driving speeds. (**a**) 200 rpm; (**b**) 300 rpm; (**c**) 400 rpm; (**d**) 600 rpm.

**Figure 12:**
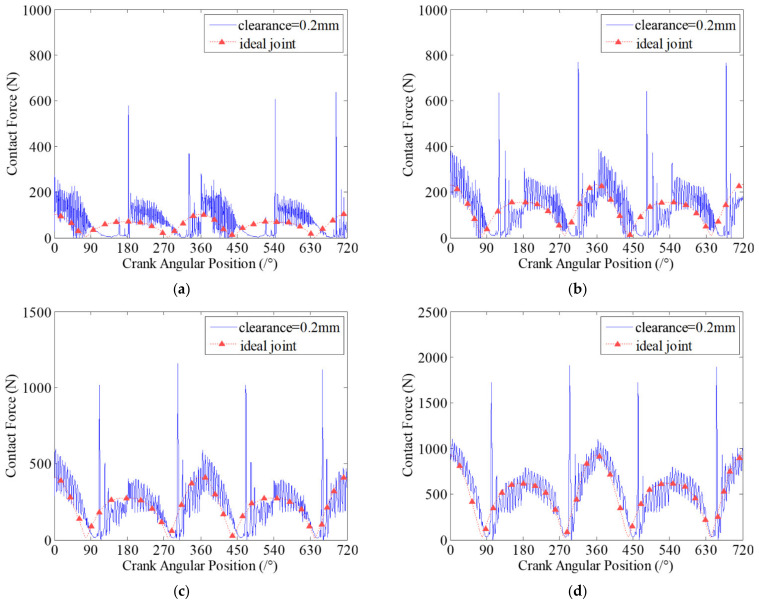
Contact forces in revolute clearance joint A for different driving speeds. (**a**) 200 rpm; (**b**) 300 rpm; (**c**) 400 rpm; (**d**) 600 rpm.

The driving speed also has a certain influence on the dynamic characteristics and wear characteristics of mechanisms with clearance joints, especially for high-speed driving mechanisms. In this section, the effects of driving speeds on the dynamic wear characteristics of clearance joint are investigated. Four case studies are presented with different driving speeds, which are 200 rpm, 300 rpm, 400 rpm and 600 rpm, respectively. The clearance size in clearance joint A and revolute joint B is 0.2 mm. The dynamic wear depths and the contact forces at clearance joint A for different driving speeds are presented in Figure 11 and Figure 12.

From Figure 11 and Figure 12, it can be clearly seen that different driving speeds present different wear levels on the dynamic wear of clearance joints. When the driving speed is higher, the contact and wear between journal and bearing in clearance joints is more severe. The reason behind this phenomenon is that the higher driving speed leads to higher contact frequency and greater contact forces. Further, it causes greater wear depth of clearance joints. When the crank speed is increased from 200 rpm to 400 rpm, and then to 600 rpm, the maximum wear depth increases by approximately 7.1 times and 24.9 times. Therefore, the drive speed has a significant influence on the wear characteristics of clearance joints in mechanisms.

## 8. Conclusions

This work studies the dynamic wear characteristics of revolute clearance joints in mechanisms using a computational methodology. The normal contact force model and the tangential friction force model are established to describe the contact-impact in clearance joints. The dynamic Archard’s wear model is established to predict the wear depth of clearance joints in mechanisms. A planar slider-crank mechanism with two revolute clearance joints is used as the implement example to perform the investigation. Different case studies for different effect factors are performed to investigate the dynamic wear characteristics of clearance joints based on the dynamic Archard’s wear model. The simulation results show that:

(1) The wear depth of clearance joints is different in each crank motion position and changes dynamically during the motion of mechanism. The wear of revolute clearance joints is not uniform, but more severe in some regions of crank position. Also, it shows that the dynamic wear depth is larger when considering two clearances compared with the wear depth when only one clearance joint is considered in the mechanism;
(2) Different clearance sizes present a similar dynamic wear phenomenon, but the wear depths of the clearance joints are obviously different. When the clearance size is smaller, the wear level is weaker and the maximum wear depth is smaller. As the clearance size increases, the wear level is more severe and the maximum wear depth increases;
(3) A higher contact stiffness coefficient can effectively reduce the wear level between the contact surfaces of the clearance joint, although the contact forces between the journal and bearing are increased as the contact stiffness coefficient increases. The reason is that as the contact stiffness coefficient increases, the ability of joint elements to resist deformation is stronger, and the contact deformation between journal and bearing decreases;
(4) Different driving speeds present different wear levels on the dynamic wear of the clearance joint. When the driving speed is higher, the contact and wear depth are more severe between the journal and the bearing in clearance joints. The higher driving speed leads to higher contact frequency and greater contact forces, which causes greater wear depth of clearance joints.

This work investigates the dynamic wear of revolute joints with clearance in mechanical systems and the proposed framework can be extended to various kinds of mechanisms with revolute clearance joints. The simulation results show that the wear in clearance joints cannot be neglected and wear characteristics analysis is the basis for life prediction of mechanisms with clearance joint wear.

## Figures and Tables

**Table 1:** Geometric and inertia properties of the slider-crank mechanism.

Component	Length (mm)	Mass (kg)	Moment of Inertia (Kg·mm^2^)
Crank	75	3.8674	9.69 × 10^3^
Connecting rod	360	0.2287	4.708 × 10^3^
Slider	-	2.3248	2.549 × 10^3^

**Table 2:** Parameters used in the dynamic simulation [26,29].

Parameter	Value
Coefficient of restitution	0.9
Coefficient of friction	0.1
Wear coefficient	5.05 × 10^−13^
Elasticity Modulus (GPa)	207
Poisson’s ratio	0.29
Crank speed (rpm)	200
Step size	0.001 s

**Table 3:** Parametric studies for different cases.

Testing Parameter	Value			
	Case 1	Case 2	Case 3	Case 4
Clearance size	0.1 mm	0.2 mm	0.3 mm	0.4 mm
Contact stiffness	1 × 10^6^ N/mm	5 × 10^6^ N/mm	1 × 10^7^ N/mm	5 × 10^7^ N/mm
Driving speed	200 rpm	300 rpm	400 rpm	600 rpm

## Data Availability

Not applicable.
